# CDK9 keeps RNA polymerase II on track

**DOI:** 10.1007/s00018-021-03878-8

**Published:** 2021-06-19

**Authors:** Sylvain Egloff

**Affiliations:** Molecular, Cellular and Developmental Biology Department (MCD), Centre de Biologie Intégrative (CBI), University of Toulouse, CNRS, UPS, 31062 Toulouse, France

**Keywords:** Cyclin T1, RNA polymerase II CTD, Transcriptional checkpoint, 7SK RNA, Promoter-proximal pausing, HIV

## Abstract

Cyclin-dependent kinase 9 (CDK9), the kinase component of positive transcription elongation factor b (P-TEFb), is essential for transcription of most protein-coding genes by RNA polymerase II (RNAPII). By releasing promoter-proximally paused RNAPII into gene bodies, CDK9 controls the entry of RNAPII into productive elongation and is, therefore, critical for efficient synthesis of full-length messenger (m)RNAs. In recent years, new players involved in P-TEFb-dependent processes have been identified and an important function of CDK9 in coordinating elongation with transcription initiation and termination has been unveiled. As the regulatory functions of CDK9 in gene expression continue to expand, a number of human pathologies, including cancers, have been associated with aberrant CDK9 activity, underscoring the need to properly regulate CDK9. Here, I provide an overview of CDK9 function and regulation, with an emphasis on CDK9 dysregulation in human diseases.

## Introduction

Cyclin dependent kinases (CDK) are serine/threonine kinases that are involved in either cell division (by promoting progression through the cell cycle), transcription by RNAPII, or both [1, 2]. Both ‘cycling CDKs’ and ‘transcriptional CDKs’ associate with a cyclin regulatory subunit that is essential for enzymatic activity. Contrary to cycling CDKs whose activity varies during the cell cycle, due to regulated cyclin degradation (this is the case for CDK1, 2, 3, 4 and 6), transcriptional CDKs keep generally constant levels of expression and activity [3]. CDK9 is the most extensively studied transcriptional CDK. Together with a cyclin T subunit, CDK9 forms the positive transcription elongation factor b (P-TEFb) that is required for efficient transcription of most RNAPII-transcribed genes. P-TEFb stimulates productive elongation through releasing promoter-proximal paused RNAPII into gene bodies [4, 5]. RNAPII pausing and release are decisive steps in the transcription cycle of the vast majority of actively transcribed protein-coding genes in both basal- and stimulus-regulated conditions, and are thought to enable a synchronous response to developmental cues and external stimuli [6]. The impact of CDK9 on RNAPII transcription extends beyond transcriptional elongation, as it also influences transcription initiation and termination, and helps RNAPII compartmentalization into phase-separated biological condensates [7]. Accurate regulation of CDK9 activity is, therefore, critical to maintain the right transcriptional output, and various pathologies have been associated with dysregulation of P-TEFb activity [8]. Here, I review the current knowledge of CDK9 function and regulation, and its involvement in human diseases.

## The general transcription factor P-TEFb

CDK9 is a nuclear protein that is expressed in all human tissues, with higher levels in terminally differentiated cells. The N-terminal region of CDK9 contains a modified PSTAIRE motif (PITALRE) which is characteristic of the CDK family kinases [2]. Two isoforms of CDK9 are expressed from the same gene and termed CDK9_(42)_ and CDK9_(55)_ based on their respective molecular weight. The 55-kDa isoform results from the use of an alternative promoter that generates a 5’-expanded first exon encoding a 117 N-terminal amino acids extension [9]. While the CDK9_(42)_ isoform is generally the most highly expressed, the relative abundance of the two proteins varies among cell lines and tissues, with CDK9_(55)_ more highly expressed in brain and liver. The ratio between the two isoforms can also change in response to intra- and extra-cellular signals [9–12]. CDK9_(42)_ and CDK9_(55)_ display the same reactivity and specificity towards substrates and seem to behave similarly [9, 11]. However, specific roles for CDK9_(55)_ in muscle regeneration, DNA repair and apoptosis have been proposed [10, 11, 13].

Both CDK9 isoforms can form heterodimeric complexes with Cyclin T1, T2a or T2b [14] but Cyclin T1 is the predominant CDK9-associated cyclin. Cyclin T2a and T2b arise from alternatively spliced transcripts generated from the same gene. They are usually present at lower levels than Cyclin T1, and are considered as minor CDK9 partners. However, Cyclin T2 may have specific functions in muscle differentiation [15–18] and is essential for mouse embryogenesis [19]. All Cyclin Ts have a characteristic cyclin box at their N-Termini, and a histidine-rich domain implicated in substrate recognition in their C-terminal regions [20, 21]. Since T2-type cyclins often display the same biochemical properties as Cyclin T1, I will consider here the Cdk9_(42)_/CycT1 heterodimer as the positive transcription elongation factor b (P-TEFb).

To be active, CDK9 needs not only to be associated with a Cyclin T, but also to be phosphorylated on the threonine 186 residue (Thr186) located in a conserved region known as the T-loop [2]. Phosphorylation of Thr186 is primarily accomplished by CDK7, another transcriptional CDK, and maybe also by self-phosphorylation [22–24]. The resolution of CDK9/Cyclin T1 crystallographic structure suggests that phosphorylation of Thr186 positions the T-loop correctly for substrate recognition and generates the specificity for the Ser/Thr-Pro motif found in major P-TEFb substrates [23].

## Functions of P-TEFb in RNAPII transcription

Transcription by RNAPII can be subdivided into three main stages: initiation, elongation and termination. During initiation, transcription factors (TFs) cooperate with coactivators, such as Mediator, to recruit the general transcription factors and RNAPII to a gene promoter to assemble the pre-initiation complex (PIC). Initiation is a very intricate process that comprises several sequential steps leading to unwinding of the DNA double helix. The transition to elongation occurs when RNAPII begins the synthesis of the RNA molecule and escapes the promoter. During elongation, either RNAPII ‘walks’ along DNA or the DNA is spooled through the polymerase [25]. However, after initiation and promoter escape, movement into the gene is generally blocked 30–60 bp downstream from the transcription start site (TSS) at an early elongation checkpoint (EEC) [6]. A failure of the RNAPII transcription complex to transition to productive elongation at this stage leads to abortive transcription. If paused RNAPII is successfully converted into elongation-competent polymerase, RNA synthesis resumes and a full-length pre-mRNA can be made. Finally, for genes encoding polyadenylated mRNA, termination occurs after transcription of the polyadenylation site (pA site) when cleavage of the RNA and changes to the RNAPII elongation complex cause termination downstream [26].

P-TEFb activity is critical for release of RNAPII that is stalled at the EEC. CDK9-mediated RNAPII pause release has recently been shown to influence the frequency of transcription initiation [27, 28]. In addition to this well-established function in early elongation control, CDK9 activity is also required for co-transcriptional processing of nascent transcripts, and for the dynamics of RNAPII close to poly(A) sites [29–31]. Thus, P-TEFb potentially regulates, directly or indirectly, all stages of the RNAPII transcription cycle.

### Regulation of RNAPII pausing during early elongation

**Fig. 1:**
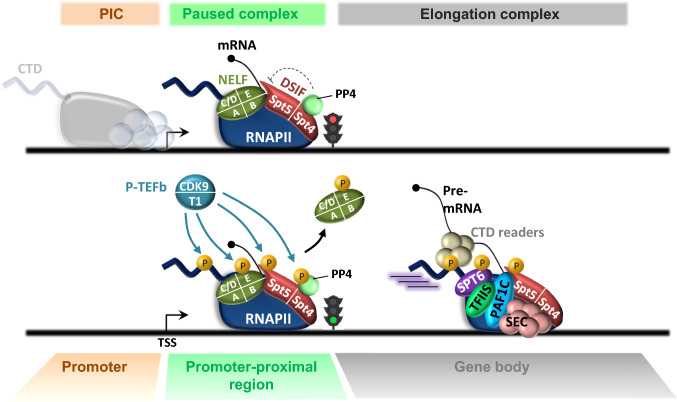
P-TEFb promotes the release of RNAPII from pause sites. Shortly after initiation, progression of RNAPII along the gene is halted by the coordinated action of NELF and DSIF, while protein phosphatase 4 (PP4) helps maintain the Spt5 subunit of DSIF in an unphosphorylated state. Once P-TEFb is recruited, it phosphorylates DSIF, NELF and the RNAPII CTD. Phosphorylation of Spt5 converts DSIF in a positive elongation factor, and NELF is evicted from the RNAPII complex. Phosphorylation of Spt5 is further reinforced by inhibitory phosphorylation of PP4. Upon pause release, accessory proteins join the elongation complex to increase RNAPII processivity and to increase the elongation rate. Phosphorylation of the CTD facilitates the recruitment of CTD reader proteins required for pre-mRNA processing. PIC: pre-initiation complex. CTD: carboxyl-terminal domain. NELF: negative elongation factor. DSIF: DRB-sensitivity inhibitory factor. TSS: transcription start site. Pre-mRNA: pre-messenger RNA

Although other factors have been implicated in pause establishment and release, promoter-proximal pausing primarily relies on the combined action of negative factors that impede RNAPII progression and positive factors that instead stimulate the transition to productive elongation [6] (Fig. 1). Shortly after initiation, RNAPII falls under the influence of two negative factors, DRB-sensitivity inducing factor (DSIF) and negative elongation factor (NELF) [32, 33]. DSIF, a heterodimer composed of Spt4 and Spt5, binds the transcription complex after RNAPII has escaped the promoter when the Spt5-binding site becomes accessible [34, 35]. Association of DSIF to the transcription machinery is also enhanced by the interaction of Spt5 with the emerging nascent RNA [36]. The four-subunit NELF complex (composed of NELF-A-B-C/D and -E) then joins the early elongation complex as it transcribes the promoter-proximal region through recognition of the RNAPII/Spt5 interface [32, 35, 37, 38]. The NELF-E subunit establishes additional contacts with the nascent RNA, where it recognizes a short consensus RNA element enriched at transcriptionally paused genes [39–41]. Whether this interaction assists in RNAPII pausing is not clear. NELF rather stabilizes paused complexes through blocking access to TFIIS [35], the activity of which is needed to rescue backtracked polymerase to allow resumption of elongation [42, 43]. In addition, NELF binding restrains RNAPII mobility and maintains the active site of paused RNAPII in a tilted conformation that is incompatible with RNA elongation [35]. Thus, DSIF/NELF-associated RNAPII is more likely to experience stable pausing, especially near the TSS, where it encounters the first nucleosome in the transcribed region [44–48]. Apart from DSIF and NELF, other factors such as SIRT6 [49], Gdown1 [50, 51], the PAF1 complex (PAF1C) [52, 53], and FACT [54] may also contribute to locking the poised RNAPII, but their direct involvement in pausing is still subject of debate.

As escape of RNAPII from pause sites requires the kinase activity of P-TEFb (Fig. 1), treatment of cells with commonly used CDK9 inhibitors such as DRB (5,6-dichloro-l-β-d-ribofuranosyl benzimidazole) or Flavopiridol (FP) results in an accumulation of RNAPII in the promoter-proximal region of most protein-coding genes [45, 55–58]. Mechanistically, three main CDK9 substrates have been identified within the early elongation complex: the two negative elongation factors, DSIF and NELF, and the carboxy-terminal domain (CTD) of RPB1, the largest RNAPII subunit. Probably the most important target of CDK9 in the pause release process is the Spt5 subunit of DSIF, the phosphorylation of which triggers dissociation of NELF from RNAPII [37, 59]. Phosphorylation of NELF itself, as well as its modification by PARP-1, may also help relieve the DSIF/NELF-mediated blockage of early elongation [60–62]. P-TEFb-mediated dissociation of NELF from the transcription complex should theoretically allow TFIIS to rescue backtracked RNAPII [35, 43] while converting Spt5 into a positive factor that remains associated with RNAPII across the gene body [58, 63]. Phosphorylation of Spt5 occurs in its repetitive C-terminal repeat region (CTR) that, like the RNAPII CTD, harbors the typical Ser/Thr-Pro motif found in most CDK substrates [59]. CDK9 strengthens SPT5 phosphorylation by also inhibiting protein phosphatase 4 (PP4), a phosphatase that stabilizes promoter-proximal pausing by keeping SPT5 unphosphorylated in the 5′ region of genes [64] (Fig. 1). After RNAPII is released, Spt5 enhances RNAPII processivity during transcriptional elongation [59, 65–69] and promotes co-transcriptional recruitment of the PAF1C, Pin1 and Tat-SF1 elongation factors, as well as RNA 3′ end processing factors [70–74]. Located near to the exiting RNA in the elongation complex [65], the phosphorylated Spt5 CTR could thus behave as a recruitment platform for a number of factors that facilitate elongation and co-transcriptional RNA processing. The P-TEFb-dependent recruitment of PAF1C, through Spt5, is of particular importance. PAF1C has been recently implicated in regulation of RNAPII pausing [52, 75] and also exerts critical functions in transcription elongation beyond the pause release step [75–78]. Structural studies indicate that NELF and PAF1C bind to RNAPII in a mutually exclusive manner [61]. P-TEFb-mediated phosphorylation of NELF and SPT5 influences the competition between PAF1C and NELF for binding of RNAPII, favoring NELF ejection and PAF1C recruitment. The prolonged presence of PAF1C within the elongation complex could prevent later reassociation of NELF, explaining why RNAPII is less prone to long-lived pausing during productive elongation. PAF1C also favours the later recruitment of CDK12 [75], another transcriptional CDK that targets the RNAPII CTD and reinforce Ser2P in the body of the gene [79].

In addition to DSIF and NELF, the carboxyl-terminal domain (CTD) of the catalytic subunit RPB1 of RNAPII is also a major target of P-TEFb. CDK9 is commonly considered as a kinase targeting serine 2 residues (Ser2) in the consensus heptapeptide Tyr^1^-Ser^2^-Pro^3^-Thr^4^-Ser^5^-Pro^6^-Ser^7^ (YSPTSPS) that comprises the RNAPII CTD, but it may also phosphorylate Ser5 and Ser7 [80, 81]. Targeted post-translational modifications of residues within the CTD basic unit, which is repeated 52 times in the human enzyme, govern the association of factors with RNAPII during the transcription cycle and coordinate transcription with mRNA processing [82, 83] [84]. Although phosphorylation of Tyr1, Thr4 and Ser7 also occurs, mass spectrometry has revealed that phosphorylation of Ser2 and Ser5 are the most abundant CTD modifications in both yeast and humans [85, 86]. The CTD is hypophosphorylated when RNAPII is recruited within the PIC, and is first phosphorylated on both Ser5 and Ser7 by CDK7, as part as the TFIIH complex, during initiation. Ser5P recruits the capping enzyme and stimulates rapid and efficient capping of the nascent RNA as soon as it emerges from RNAPII, while Ser7P, helped by Tyr1P, primes the CTD for subsequent Ser2 phosphorylation by P-TEFb [80, 81]. Recruitment of P-TEFb to the transcriptional machinery also relies on prior citrullination of a specific arginine residue (R1810) located on repeat 31 of the mammalian RNAPII CTD [87]. Thus, phosphorylation by CDK9 can only take place after prior decoration of the CTD, perhaps to ensure effective initiation and mRNA capping. Ser2P is considered as a hallmark of productive elongation and is markedly enriched across gene bodies, with a peak at the 3′ end of genes, over terminator regions. Such high level of Ser2P likely reflects the cooperativity of CDK9 and CDK12 at the 3′ end of genes (see below). Phosphorylation of the CTD of elongating RNAPII enables recruitment of additional CTD-associating proteins and positions them near the site of transcription. Factors that recognize the Ser2P mark mainly encompass chromatin-remodeling enzymes and RNA processing complexes [88–95]. Accordingly, CDK9 activity is critical for efficient splicing, and for the recruitment of polyadenylation factors at the 3’ ends of RNAPII-transcribed genes [88, 89, 91]. Recent data indicate that P-TEFb also phosphorylates the RNAPII CTD linker region that connects the catalytic core of the enzyme to the CTD during the transition to productive elongation and by doing so, promotes recruitment of the SPT6 histone chaperone to the elongation complex [61, 96, 97]. Spt6 supports efficient transcription elongation through stimulating disassembly and reassembly of nucleosomes during RNAPII progression [98–101]. Finally, P-TEFb is often incorporated into large complexes, called Super Elongation Complexes (SECs), which comprise multiple transcription elongation factors [102]. When recruited to genes, the SEC simultaneously provides several activities that increase RNAPII processivity and stimulate RNA synthesis [103–105].

By counteracting the effects of negative elongation factors, decorating the RNAPII CTD, and supplying RNAPII with key elongation factors, P-TEFb not only releases the DSIF/NELF-mediated elongation barrier but also equips the polymerase to ensure the efficiency of downstream transcriptional and co-transcriptional events. Modulating pause release by P-TEFb is, therefore, an elegant way to fine-tune gene expression to produce the right transcriptional output.

### Functional significance of RNAPII pausing

**Fig. 2:**
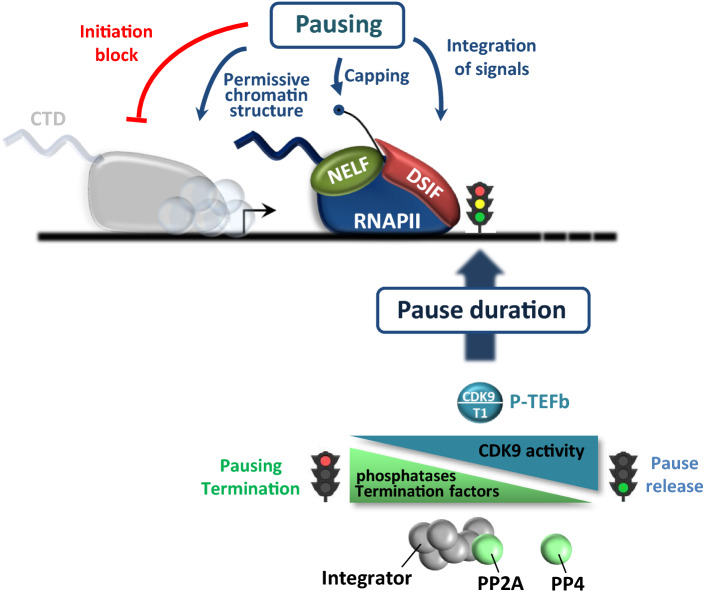
Functions of promoter-proximal pausing and regulation of pause duration. Promoter-proximal pausing limits the rate of new initiation by RNAPII, facilitates capping of the nascent pre-mRNA and provides a window of time for integration of external signals or stimuli. Pause duration is tightly regulated and can vary from one gene to another. The residence time of RNAPII at pause sites is driven by the balance between P-TEFb recruitment, phosphatase activities and recruitment of termination factors such as Integrator, which can promote premature termination. CTD: carboxyl-terminal domain. NELF: negative elongation factor. DSIF: DRB-sensitivity inhibitory factor. PP: protein phosphatase

In all metazoans, RNAPII distribution along genes shows an increased density just downstream of the TSS [106, 107]. As this profile primarily results from an equilibrium between RNAPII recruitment, initiation, and pause release into either productive elongation or termination (see below), it suggests that the rate of pause release is generally lower than the rate of initiation. While the kinetics of these two regulatory stages were long considered to be independent of each other, recent studies have highlighted important connections between pausing and the frequency of transcription initiation. In both *Drosophila* and human cells, an inverse correlation between the levels of promoter-proximally paused RNAPII and of newly initiating RNAPII has been observed, with highly paused genes displaying lower initiation rates [27, 28]. Accordingly, increasing RNAPII pausing through inhibition of CDK9-mediated pause release generally results in a concomitant decrease of transcription initiation (Fig. 2). The mechanisms underlying this relationship are not clearly defined, but the presence of RNAPII at pause sites may sterically impede subsequent initiation by another polymerase and limit the frequency of re-initiation [28]. However, RNAPII pausing itself also have a positive effect on transcription initiation as accumulation of RNAPII in promoter-proximal regions creates a permissive chromatin structure that allows factors to access the promoter [108–110]. Indeed, RNA interference-mediated depletion of NELF reduces RNAPII pausing, increases nucleosome occupancy at many promoters and decreases transcription initiation [109, 111] (Fig. 2). Thus, the emerging view is that CDK9-mediated release of pausing stimulates both productive elongation (by promoting progression of RNAPII into gene bodies) and initiation (by reducing RNAPII occupancy at pause sites), which could have synergistic effects on the amount of transcription.

Promoter-proximal pausing was also proposed to provide a window of time for co-transcriptional capping of the nascent RNA to occur [112]. As addition of a 5′ methyl-7-Guanosine (m7G) cap protects the 5′ end of nascent mRNA from degradation [113], it is important that this happens as soon as possible after the RNA 5′ end emerges from RNAPII. Phosphorylation of Ser5 residues by CDK7 early in the transcription cycle turns the RNAPII CTD into a landing pad for the capping machinery [114–117]. The capping enzyme can also access the nascent RNA through interaction with the Spt5 subunit of DSIF, that also stimulates capping [118–120]. Capping appears to start when the transcript is only 20–25 nucleotides long [121] and may occur progressively as RNAPII moves towards the pause region, raising the possibility that nascent RNAs are already capped when NELF-mediated pausing occurs. NELF has recently been shown to recruit the cap-binding complex (CBC) to the transcription machinery [122, 123] to interact with the cap to protect the capped RNA from decapping [124]. The CBC may in turn help to recruit P-TEFb [91]. Thus, DSIF/NELF-mediated RNAPII pausing might function as a quality-control process ensuring that mRNA are properly protected at their 5′ end before RNAPII enters productive elongation (Fig. 2).

On some genes, RNAPII is poised for activation and function as scaffold for integration of environmental and developmental signals [125] (Fig. 2). Promoter-proximal pausing could provide storage of transcriptionally engaged, ‘ready to go’ polymerases, awaiting activation stimuli for rapid and synchronous release of RNAPII into productive elongation [126, 127]. Several key biological processes may rely on poised RNAPII for gene activation, including the heat shock response [128, 129], early embryonic development [126, 130, 131], inflammatory responses [132], the DNA damage response [133, 134] and hormone-regulated signaling [131, 135]. Keeping the polymerase in a state of readiness could thus allow transcription of biologically relevant genes to rapidly respond to external/developmental cues [132, 136]. The early elongation checkpoint thus adds a further possibility to regulate transcription before RNAPII has gone very far [6] and allows synergic or antagonists signals to be integrated during the early steps of transcription.

On the majority of genes, paused RNAPII appears to be quite stable, with a residence time between 5 and 10 min [28, 45, 137]. However, RNAPII can sometimes remain engaged on a gene for 30–60 min while awaiting signals for pause release [28, 56, 137]. It is likely that poor recruitment of P-TEFb to these genes is responsible for increased paused RNAPII stability. Since paused RNAPII blocks new initiation, prolonged pausing may make a gene refractory to activation [138]. In contrast, a significant proportion of genes harbors very unstable promoter-proximally paused RNAPII that is quickly dissociated from the DNA template through premature termination [110, 139, 140]. The rapid turnover of RNAPII at these genes prevents the polymerase from entering productive elongation, thus repressing gene activity. The instability of this paused RNAPII is due to the recruitment of the integrator complex, whose endonucleolytic activity triggers cleavage of nascent mRNA transcripts and promoter-proximal termination [139]. Recent findings showed that two protein phosphatases, PP2A and PP4, can keep Spt5 unphosphorylated in the promoter-proximal region, thus promoting pausing by counteracting CDK9-mediated phosphorylation [64, 141, 142]. A non-canonical PP2A enzyme, associated with the Integrator complex, may also dephosphorylate the RNAPII CTD [142, 143]. Thus, competitive recruitment of P-TEFb, phosphatases and termination factors during early elongation likely determines the stability and the fate of paused RNAPII (Fig. 2).

### CDK9 regulates transcription beyond pausing

**Fig. 3:**
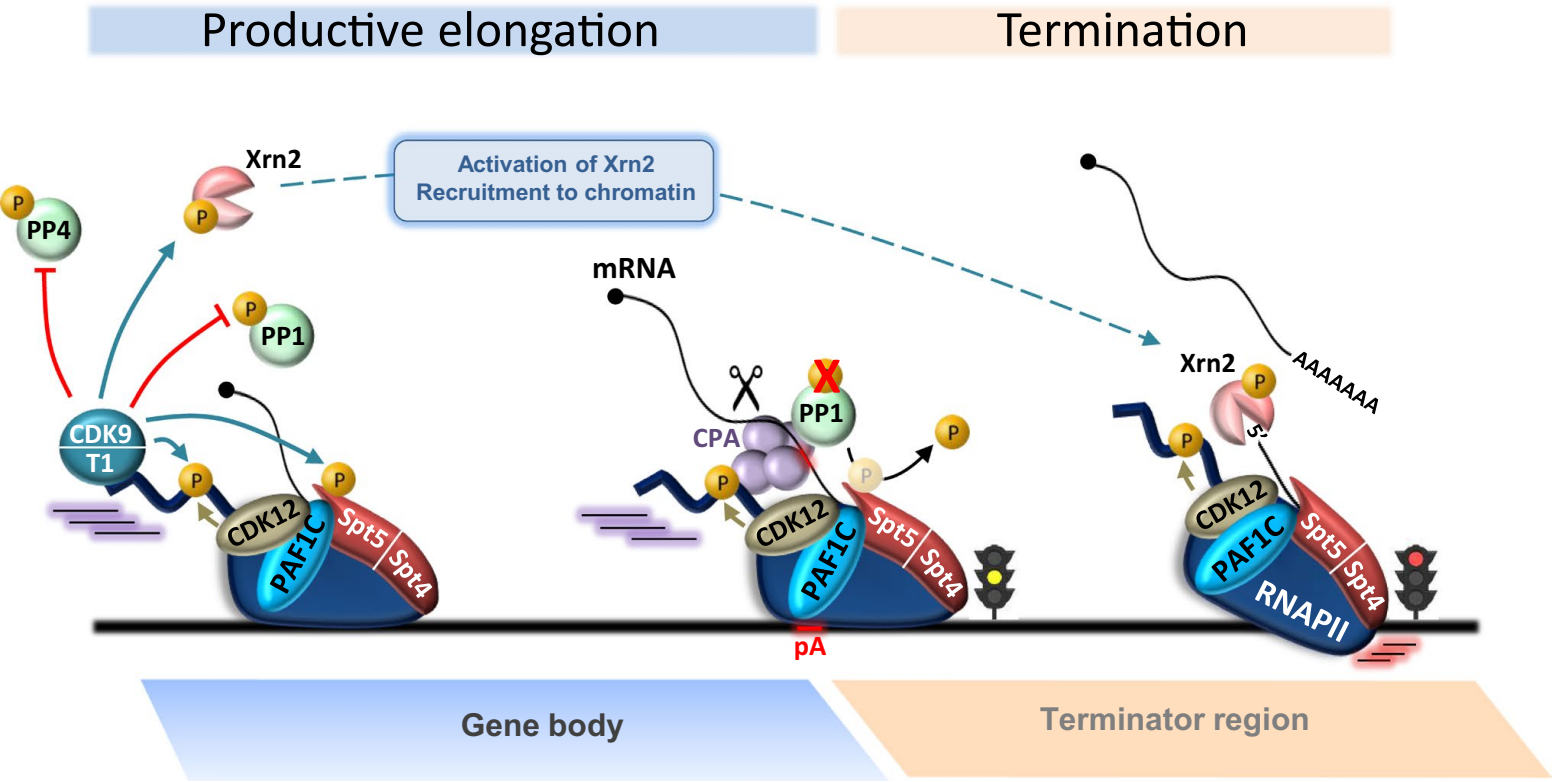
CDK9 regulates transcription across the polyA site. During elongation, CDK9 phosphorylation inactivates PP4 and PP1 phosphatase activities, thus maintaining high levels of Spt5 phosphorylation. Once RNAPII transcribes through the polyA (pA) site, Ser2P recruits the cleavage and polyadenylation machinery (CPA), which is critical for proper termination. Concomittantly, CDK9 activity drops and PP1 becomes active, leading to dephosphorylation of SPT5. Unphosphorylated Spt5 acts as a brake for RNAPII that becomes a preferential substrate for CDK9-activated Xrn2 exonuclease. PP: protein phosphatase. CPA: cleavage and polyadenylation factors

Genome-wide nascent transcription analysis techniques such as global run-on sequencing (GRO-seq) confirm that RNAPII stalls close to the TSS of the majority of genes after addition of CDK9 inhibitors [29, 45, 84, 144]. As visualized on long genes, RNAPII that has already passed the EEC can continue to transcribe for long distances even if CDK9 activity is inhibited, demonstrating that sustained CDK9 activity is not mandatory for transcription through gene bodies [29, 45, 145]. However, when RNAPII reaches the end of the transcribed region, it terminates transcription prematurely at the end of the last exon, near the poly(A) site, at what has been termed a poly(A)-associated elongation checkpoint [29]. Phosphorylation of Spt5 by CDK9 may be critical to negotiate this checkpoint, as Spt5 association with the poly(A) region is drastically reduced by CDK9 inhibition. Indeed, CDK9-mediated phosphorylation of Spt5 is normally erased by protein phosphatase 1 (PP1) when RNAPII passes the poly(A) site [30, 146]. This drop in Spt5 phosphorylation is followed by RNAPII slowing down downstream of the poly(A) site. RNAPII then accumulates downstream of the pA site and becomes heavily phosphorylated on Ser2, thus facilitating the recruitment of factors involved in mRNA 3′ end processing and termination [79, 147, 148] (Fig. 3). CDK9 activity is required to maintain the phosphorylation status of Spt5 upstream of the poly(A) site, most likely by preventing Spt5 dephosphorylation by PP1. Indeed, PP1 is a target of CDK9 in both yeast and human [30, 31] and this phosphorylation is inhibitory [30, 64]. Thus, CDK9 neutralizes PP1 activity during elongation, thereby preserving Spt5 phosphorylation until RNAPII reaches the poly(A) site. In turn, Spt5 phosphorylation prevents premature termination by maintaining rapid RNAPII elongation, and perhaps by delaying the action of termination factors until poly(A) site selection has occurred [147, 149].

CDK9 also directly regulates termination by promoting recruitment and activation of the 5′ to 3′ “torpedo” exonuclease Xrn2 via an RNAPII CTD-independent mechanism [31]. Phosphorylation of Xrn2 by CDK9 appears critical for chromatin localization of Xrn2 and efficient termination in human cells [31]. In the torpedo termination model, the pre-mRNA is cleaved after RNAPII transcribes the poly(A) site, and Xrn2 degrades the downstream RNA fragment co-transcriptionally until it collides with RNAPII and dissociates it from the DNA template [26, 150]. As the torpedo model is based on the ability of Xrn2 to degrade nascent RNA faster than RNAPII synthesizes it, polymerases slowly elongating downstream of the poly(A) site are easily caught by the activated Xrn2, facilitating RNAPII dissociation and transcription termination (Fig. 3).

CDK9, therefore, plays an important role in regulating the elongation to termination switch, a step that is more strictly regulated than initially thought. The addition and removal of phosphate by the CDK9/PP1 pair is critical for Spt5-dependent regulation of RNAPII dynamics during its entry to the terminator region. The mechanism by which PP1 becomes active once RNAPII has transcribed through the poly(A) remains unknown, but it could result from targeted dephosphorylation of PP1 and/or a drop in CDK9 activity. In yeast, the CDK9 homolog dissociates from the elongation complex upstream of the poly(A) site [151], which could provide an explanation for PP1 activation. Recognition of the poly(A) site is also required for the change in Spt5 phosphorylation, suggesting that assembly of a 3′ processing complex on the poly(A) site and/or cleavage of the nascent RNA is involved in PP1 activation [146] (Fig. 3). Interestingly, the yeast cleavage and polyadenylation factor (CPF) contains a PP1 holoenzyme [152, 153], and could target PP1 to the transcription machinery at the 3′ end of genes.

Additional targets of P-TEFb include the AFF1 and AFF4 subunits of the SEC [154, 155], the androgen receptor [156], pirh2 [157], pRB [158] and p53 [159, 160]. P-TEFb also phosphorylates the chromatin regulators histone H1, UBE2A, BRG1 and Tip60 [161–164]. A recent in vitro study aimed at identifying the full repertoire of CDK9 targets revealed numerous additional factors (more than 100), half of which are implicated in transcription and/or RNA metabolism [31]. Decker et al. (2019) used a CDK9 analog-sensitive human cell line to identified 120 phosphosites which were quantitatively decreased upon CDK9 inhibition and confirmed that the major CDK9 substrates were transcription- and RNA processing-associated factors [165]. Potential new P-TEFb targets include splicing factors, chromatin modifiers, pause release factors and proteins implicated in RNA quality control. One should, therefore, expect future studies to assign additional function(s) to CDK9 and further expand the mechanisms by which it regulates RNAPII transcription.

### CDK9 and CDK12: distinct CDKs with the same CTD target

It is important to note that CDK9 is not the only Ser2P kinase, as two other ‘transcriptional’ CDKs, CDK12 and CDK13, also contribute to this critical CTD modification [79, 166–170]. While both CDK12 and CDK13 are bound to cyclin K and target the CTD in vivo, most studies have focused on CDK12’s function, mainly because it has emerged as an important player in human cancers [171, 172]. Depletion or inactivation of CDK12 leads to altered CTD phosphorylation in both *Drosophila* and human cells [166–168, 173–175]. Its genomic distribution suggests that CDK12 functions on elongating RNAPII, downstream of CDK9, and contributes to the increased Ser2P signals towards the 3′ end of genes [79, 176] (Fig. 3). However, a number of studies have also identified Ser5 of the CTD as a target of CDK12 [168, 173, 175, 177]. CDK9 and CDK12 are close collaborators, as recruitment of CDK12 depends on CDK9 activity [75]. It makes the relative contribution of each kinase to Ser2P difficult to appraise. Although the CTD appeared to be the main substrate of CDK12 and CDK13, the exact function of these CDKs has long remained unclear [171]. Despite its localization on actively transcribed genes genome-wide [178], CDK12 was shown to specifically promote expression of subsets of human genes, i.e., DNA damage/DNA repair genes [167, 174, 178, 179] and DNA replication genes [173]. However, genome-wide nascent transcription analyses indicate that CDK12 is a global regulator of RNAPII processivity, required for efficient elongation of transcription of the vast majority of RNAPII-transcribed genes [168, 169]. CDK12 is required for stable association of PAF1C and Spt6, two critical elongation factors, with the elongating RNAPII machinery [168]. Maintenance of high RNAPII elongation rates by CDK12 may be critical to prevent premature transcription termination, particularly on long genes [173, 179–181]. Mechanistically, CDK12 activity suppresses cleavage at intronic polyadenylation sites, restricting production of truncated mRNAs. Since DNA damage response genes tend to harbor a higher density of intronic polyadenylation sites than other genes [179], loss of CDK12 primarily affects expression of this class of transcripts. CDK13 displays substantial redundancy with CDK12 for boosting RNAPII processivity [169], but is also specifically required for proper expression of small non-coding RNA genes, by activating RNA processing rather than transcription [174]. Identification of CDK12-associated proteins and CDK12 targets supports a broad function for this kinase in transcription, and in co-transcriptional pre-mRNA processing [174, 179, 182, 183]. Accordingly, loss of CDK12 has been shown to impede 3′ end processing of *C-FOS* and *C-MYC* genes [79, 184], likely reflecting defective Ser2P-mediated recruitment of RNA processing factors. Thus, the primary function of CDK12 (and CDK13) may be to maintain a sustained level of Ser2 phosphorylation on the elongating RNAPII (Fig. 3). By doing so, it helps RNAPII to reach maximum elongation rates to suppress usage of cryptic polyadenylation, which is particularly important on long and polyA site-rich genes [173, 179], but it also stimulates efficient co-transcriptional RNA processing.

CDK9, CDK12 and CDK13, therefore, all cooperate to ensure efficient elongation, as they all target Ser2 residues within the CTD and contribute to Ser2P [169]. Both CDK9 and CDK12 have been proposed to be key players in RNA processing, but it is difficult to disentangle the precise function of each CDK. As blocking CDK9 activity will affect subsequent CDK12 recruitment to the transcription machinery [75], the resulting effects could reflect the loss of both CDK activities. The use of pharmacological inhibitors has proven effective to block CDKs activity and study their functions. Although Flavopiridol and DRB inhibit CDK9 at lower concentrations than CDK12/CDK13 [177], complete inhibition of CDK9 could still significantly affect CDK12 and CDK13 activities, and potentially other kinases as well [182]. The recent development of more selective CDK9 inhibitors [185] and CRISPR/Cas9-engineered cell lines, which allow selective inactivation of CDK9, CDK12 and CDK13 by ATP analogs [27, 165, 168, 169, 173, 175], will help to decipher the function of each individual CDK in CTD phosphorylation and RNAPII transcription.

### P-TEFb promotes formation of compartmentalized ‘elongation-associated’ foci

Microscopy studies have revealed dynamic foci within the nucleus that are enriched in RNAPII, termed transcription ‘factories’ or ‘condensates’ [7, 25, 186, 187]. These membrane-less compartments can form by liquid–liquid phase separation and rely on cooperative interactions between intrinsically disordered protein regions (IDRs) [188]. Although their components can continuously interact with the neighboring environment, the local concentration of factors is high within condensates, favoring intermolecular interactions and efficient biochemical reactions. Some transcription (co)factors use their disordered transactivation region to form such condensates at sites of active transcription [186, 187, 189–192]. They recruit and trap RNAPII through its intrinsically disordered hypophosphorylated CTD, providing a mechanism for efficient recruitment of RNAPII to both enhancers and promoter regions to support high rate of transcription initiation [190, 192, 193]. Importantly, CTD phosphorylation by CDK7 weakens interaction with activators and disrupts phase separation as RNAPII escapes the promoter [193, 194]. Thus, transcriptional condensates could be seen as dynamic hubs that enable efficient regulation of RNAPII transcription.

**Fig. 4:**
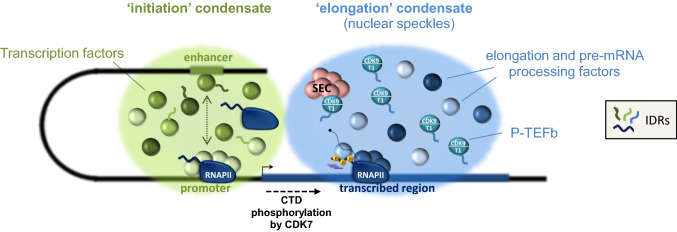
Cyclin T1 promotes the formation of ‘elongation condensates’ through liquid–liquid phase separation. Intrinsically disordered regions (IDRs) of transcription factors trigger the formation of ‘initiation’ condensates at gene promoters and enhancers and favour recruitment of RNAPII and gene activity. The Cyclin T1 histidine-rich region (HRD) organizes a phase-separated environment that promotes incorporation of P-TEFb into another type of condensate associated with transcription elongation (or nuclear speckles). These condensates simultaneously concentrate pause release, elongation and processing factors. CTD phosphorylation by CDK7 relocates RNAPII from ‘initiation’ to ‘elongation’ condensates, providing RNAPII with critical activities required for the next stage of the transcription cycle. CTD: carboxy-terminal domain

Recent data indicated a function for the histidine-rich domain (HRD) of cyclin T1 in creating another type of transcription condensate that instead of being connected to transcription initiation, is associated with elongation [7, 20] (Fig. 4). The low-complexity HDR of Cyclin T1 is located within a broader IDR in its C-term domain and it was shown to support both interaction with the CTD of RNAPII and phase separation [20, 21]. Consistently, Cyclin T1 lacking the HDR is unable to form droplets, displays a reduced activity towards the CTD as well as a shorter residence time on chromatin. The Cyclin T1 HRD may organize a phase-separated environment leading to local accumulation of RNAPII together with P-TEFb, thereby stimulating efficient hyperphosphorylation of the CTD and elongation. Recent data showed that Cyclin T1 can phase separate in the presence of SEC components [195]. These ‘elongation-associated’ condensates can be visualized in cells by imaging techniques and correspond to the nuclear speckles [20, 194, 196], which also contain a number of additional elongation and pre-mRNA processing factors [197, 198] (Fig. 4). Incorporation of the CTD within these compartments is enhanced upon pre-phosphorylation by CDK7 [20, 194], suggesting that CTD phosphorylation on Ser5/Ser7 relocates RNAPII from initiation-associated to elongation-associated condensates during early elongation (Fig. 4). Thus, ‘elongation-associated’ condensates could simultaneously concentrate pause release, elongation and co-transcriptional processing factors around transcriptionally engaged RNAPII complexes to favor robust transcription through chromatin. The fact that hypo and hyper-phosphorylated forms of RNAPII occupy contiguous nuclear locations [199] supports the hypothesis that RNAPII shuttles from ‘initiation’ to ‘elongation’ condensates, which would physically separate initiation- and elongation-related factors for increased efficiency. More work is needed to understand the impact of phase separation on each step of the transcription process. Given their reversible nature, future studies should also establish whether the appearance and disappearance of transcription condensates plays a role in regulating transcription programs, such as responses to developmental stimuli, viral infection or external cues.

## Regulation of CDK9 activity

Since P-TEFb is a master regulator of RNAPII transcription, controlling its nuclear activity is of particular importance. Two main regulatory mechanisms together govern the rate of CDK9-mediated release of paused RNAPII. First, CDK9 cellular availability can be restrained by the 7SK inhibitory snRNP. Second, P-TEFb has to be efficiently targeted to gene promoters through interaction with transcription factors/complexes.

### The 7SK snRNP: a reservoir of CDK9 activity in the nucleoplasm

Binding of cyclin-kinase inhibitors (CKI) is a common way to regulate CDKs activity [200]. CDK9 is, however, the only known CDK that is regulated by an RNA-containing CKI. In human cells, a significant fraction of P-TEFb is found in a large, catalytically inactive complex, associated with the 7SK small nuclear (sn)RNA, MePCE, LARP7 and HEXIM1/2 proteins [5, 201]. Within this ribonucleoparticle (RNP), the 7SK methylphosphate capping enzyme (MePCE) and the La-related Protein 7 (LARP7) stably associate with the 5′ and 3′ ends of the 331 nucleotides-long 7SK snRNA, respectively, and are both essential for 7SK snRNA stability and snRNP integrity [202–204]. Protected by the core snRNP proteins, the 7SK snRNA provides the structural RNA scaffold for the formation of the P-TEFb inhibitory complex. Its 5′ terminal hairpin forms a docking site for a homo- or hetero-dimer of HEXIM1/2 (Hexamethylene Bisacetamide-inducible Protein), binding of which relieves an auto-inhibitory conformation of HEXIM proteins and exposes their otherwise inaccessible P-TEFb binding surface [205–208]. 7SK-dependent binding of HEXIM1 to both CDK9 and Cyclin T1 prevents substrates from accessing the CDK9 catalytic site, thus providing a plausible mechanism for P-TEFb inhibition [209, 210]. Additional interaction between LARP7 and CDK9 may further reinforce the assembly of 7SK/P-TEFb RNP [211, 212]. Although some structural hints have been provided for binding of MePCE and Larp7 to 7SK [213, 214], the structure of the complete 7SK/P-TEFb snRNP has not yet been resolved. Importantly, only Thr186-phosphorylated CDK9 is successfully incorporated within the inhibitory RNP, ensuring that only activated, ready to use P-TEFb is sequestered into the 7SK/P-TEFb snRNP [215, 216].

**Fig. 5:**
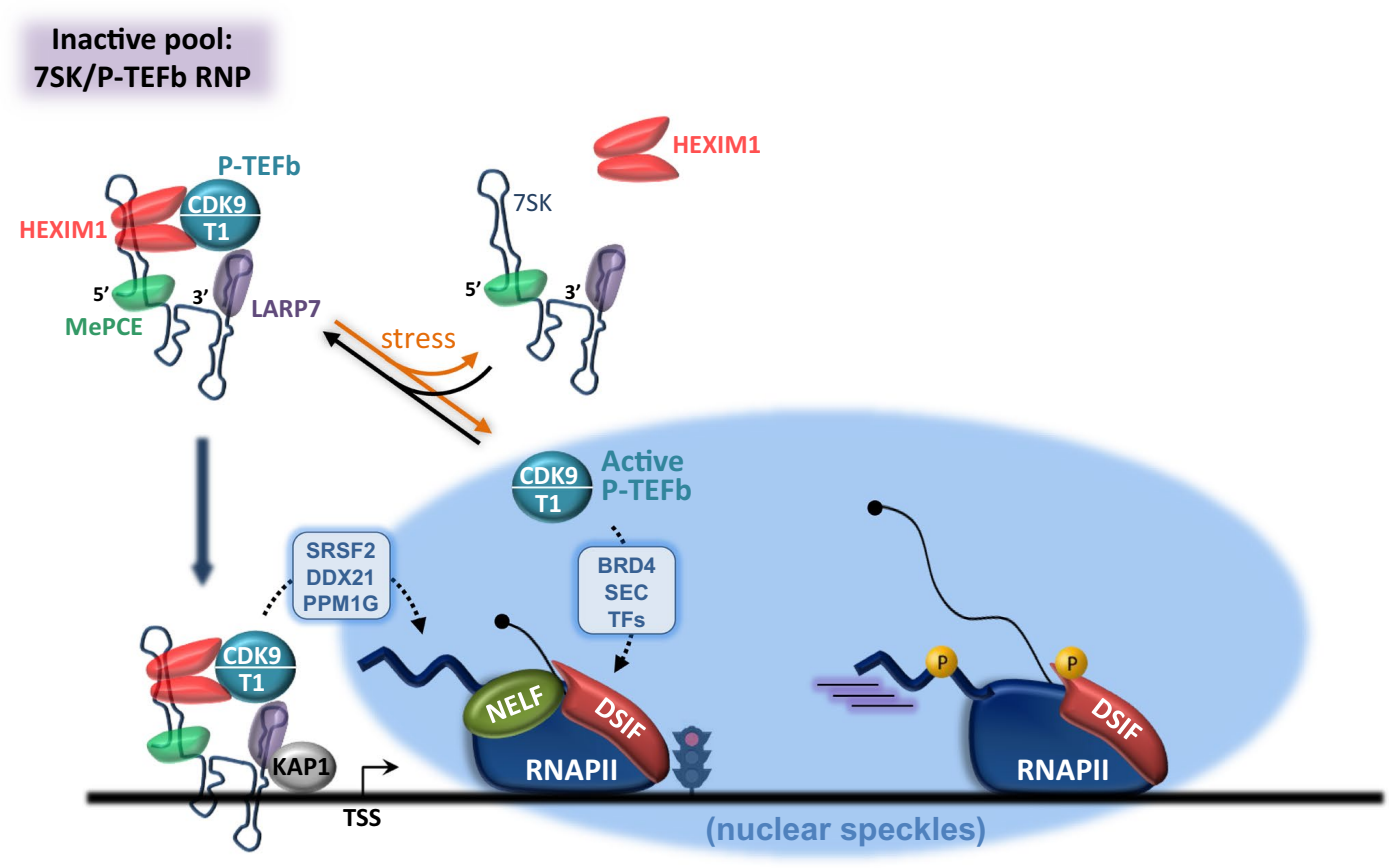
Regulation of CDK9 availability by the 7SK snRNP. In the nucleoplasm, a fraction of P-TEFb is sequestered into the 7SK/P-TEFb snRNP, where CDK9 activity is inhibited by HEXIM1 in a 7SK-dependent manner. After cellular stress, disassociation of the 7SK/P-TEFb snRNP releases active P-TEFb, which can be loaded onto target genes by BRD4, Super Elongation Complexes (SEC) or gene-specific transcription factors (TF). The 7SK/P-TEFb snRNP is also directly targeted to gene promoters by KAP1. Factors such as SRSF2, DDX21 or PPM1G can activate P-TEFb from chromatin-anchored 7SK/P-TEFb snRNP in the vicinity of the RNAPII complex for ‘on site’ transcriptional activation. Note that only the active form of P-TEFb can be incorporated into transcription-associated condensates

The amount of P-TEFb kept ‘in custody’ by the 7SK RNP is dictated by the overall transcriptional need, as well as the proliferative state of the cell [217]. For instance, 50% of P-TEFb is inactivated in actively growing Hela cells, but 7SK binds up to 90% of P-TEFb in human primary blood lymphocytes [218–221]. While it considerably restricts CDK9 availability under normal conditions, the P-TEFb/7SK RNP rapidly disassembles in response to cellular stress, robustly increasing CDK9 activity [220, 221] (Fig. 5). Thus, the 7SK/P-TEFb snRNP acts as CDK9 storage complex that can release active P-TEFb under stress conditions without requiring new protein synthesis. Stimuli that have been shown to trigger extraction of P-TEFb from its snRNP repressor include transcription blockade, DNA damage, viral infection and activation of various signaling pathways [201, 220–223]. Acetylation of cyclin T1 [224] and ubiquitinylation and phosphorylation of HEXIM1 [225, 226] have all been shown to shift the P-TEFb equilibrium towards the active form at the expense of the inactive form. Erasure of CDK9 T-loop phosphorylation also disassembles the 7SK RNP [227–229], although in this case the released CDK9 needs re-phosphorylation on Thr186 to become fully active. Transient rearrangement of 7SK RNA structure [230], destabilization of 7SK RNA [231–233], and binding of various RNA-binding proteins to the 7SK RNP [207, 234–240] may represent alternative/cooperative mechanisms to release P-TEFb. Nuclear speckles could be sites of active assembly/disassembly of inactive P‐TEFb complexes [196] and transition from the inactive to the active form may involve phase separation too [195]. Indeed, CyclinT1 is unable to phase separate together with HEXIM1 and the P-TEFb/7SK snRNP is widely dispersed in the nucleus, suggesting that incorporation within the 7SK snRNP prevents CDK9/Cyclin T1 translocation into transcription-associated condensates.

Retention of P-TEFb within the 7SK RNP is a way of separating and storing pre-activated CDK9 in the nucleoplasm, away from the transcription complex. However, recent studies have raised the intriguing possibility that the 7SK/P-TEFb regulatory RNP can also be anchored to chromatin, where it can activate RNAPII transcription locally through ‘on site’ release of active P-TEFb [241] (Fig. 5). First described as a gene-specific mechanism [228, 242], positioning of the 7SK/P-TEFb RNP on chromatin was next extended to more than 15,000 human genes, where LARP7, CDK9, HEXIM1 and RNAPII co-localize at promoter regions [238, 243]. Widespread 7SK/P-TEFb RNP anchoring to chromatin is mediated by the Kruppel-associated box-interacting protein KAP1 [243], while on site disassembly of the RNP can be driven by PPM1G, SRSF2, DDX21, WDR43 or JMJD6 [228, 232, 233, 236, 238, 244]. However, analyses of 7SK RNA occupancy by chromatin isolation by RNA purification (ChIRP) argue against stable 7SK RNP association with promoter regions, with accumulation of 7SK snRNA being mainly detected on super-enhancers [233, 245], snRNA genes [246] and within the whole transcribed regions of some human protein-coding genes [245, 247]. Microscopy studies also failed to detect 7SK association with a stably integrated active gene [248]. Instead, 7SK RNA transiently associates with the locus upon transcriptional shutdown and was proposed to displace P-TEFb, raising the possibility that 7SK rather serves as a dynamic P-TEFb carrier ensuring its delivery and/or removal from genes. Thus, it is not clear yet whether the 7SK RNP complex is stably anchored or only loosely associated with promoters, and how widespread this mechanism is.

### Targeted delivery of active P-TEFb

The proportion of active and inactive forms of P-TEFb changes according to the proliferative/differentiation state of cells and transcriptional need [217, 249]. Even if the 7SK snRNP is capable of anchoring inactive CDK9 to genomic loci, recruitment of P-TEFb in its active form remains the main CDK9 delivery pathway. The level of P-TEFb brought to the gene will dictate the average pause duration and the fate of poised RNAPII. If active P-TEFb is efficiently recruited by a promoter, the duration of the pause will be shorter and more RNAPII will successfully enter productive elongation. Appropriate P-TEFb recruitment is, therefore, critical to establish specific gene expression programs.

A number of cellular activators can help to target CDK9 to specific DNA or RNA sequences [4]. For example, the bromodomain protein 4 (BRD4) is an important partner of active CDK9 as it directs free P-TEFb to promoters and enhancers through interaction with acetylated histones and/or the Mediator complex [233, 250]. BRD4 directly interacts with Cyclin T1 but the BRD4/P-TEFb interaction also relies on CDK9 phosphorylation on Ser175 [250, 251]. Most of the P-TEFb that is not sequestered in the 7SK/P-TEFb snRNP associates with BRD4 and P-TEFb freshly released from the 7SK snRNP joins the BRD4 pool [240, 252]. Accordingly, RNAi-mediated depletion or chemical inhibition of BRD4 has a profound impact on P-TEFb recruitment, RNAPII transcription elongation and gene expression [240, 250, 253–255]. However, the use of a recently developed BRD4 degron system showed that rapid depletion of BRD4 reduces RNAPII release into gene bodies without affecting P-TEFb occupancy on chromatin [256]. Thus, BRD4 may not be strictly required for targeting P-TEFb to genomic regulatory regions, but may largely function as an activator of chromatin-bound P-TEFb [257]. For instance, BRD4 could collaborate with JMJD6 (Jumanji C-domain-containing protein 6) at enhancer regions to evict P-TEFb from the 7SK inhibitory complex, through demethylation of 7SK cap and/or targeted degradation of MePCE [232, 233]. The resulting ‘on site’ activation of CDK9 would then facilitate pause release and gene activity. However, additional studies will be required to clarify the function of BRD4 in CDK9 recruitment and in P-TEFb-mediated activation of productive elongation.

While association with Brd4 may represent a general way to recruit CDK9 to genes, a number of specific transcription factors (TF) can interact with and deliver active P-TEFb to to their respective target genes to facilitate transcriptional activation under basal and/or signal regulated conditions. TFs targeting P-TEFb to chromatin in a gene-specific manner include NF-kB [258], c-myc [58], MyoD [16], STAT3 [259] or MEF2 [260]. P-TEFb can also be incorporated into larger Super Elongation Complexes (SEC or SEC-like) together with other elongation factors, such as the eleven-nineteen Lys-rich leukemia (ELL) family members (ELL1/2/3), AF9, ENL, EAF1/2, AFF1 and AFF4 [102]. The SEC stimulates RNAPII processivity very efficiently [104, 105, 124] and helps to recruit CDK9 to active promoters and enhancers through interaction with the Mediator [261], PAF1C [262] or the Integrator complex [263]. Thus, numerous transcription factors and macromolecular complexes orchestrate the coordinated delivery of CDK9 to precise chromatin locations to regulate both basal and activated transcription.

## CDK9 and diseases

**Fig. 6:**
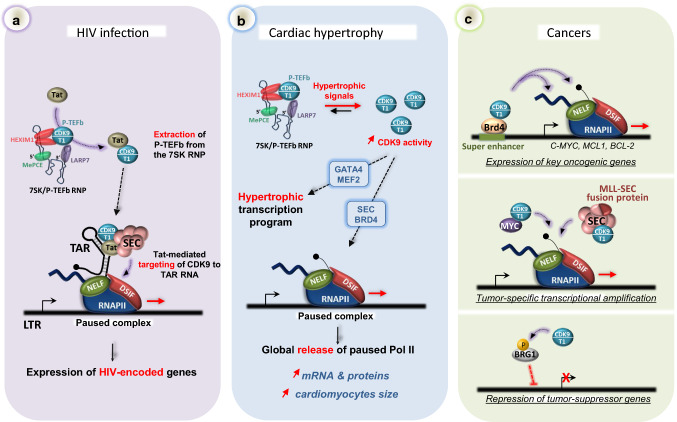
Involvement of CDK9 in human disease. **a** CDK9 is a primary target of HIV-1. The virus-encoded protein Tat extracts P-TEFb from the 7SK/P-TEFb RNP and increases the level of active P-TEFb in infected cells. P-TEFb is then tethered to the viral genome through interaction with the TAR structure that forms at the 5′ end of the viral nascent RNA and serves as a landing pad for the P-TEFb/Tat/SEC complex. Once recruited, P-TEFb releases paused RNAPII in the same way as it does on host protein-coding genes. **b** In cardiomyocytes, hypertrophic signals shift the equilibrium towards the P-TEFb active form. The resulting elevated CDK9 activity leads to the establishment of a hypertrophic transcription program and a global release of paused RNAPII into gene bodies. Elevated transcription leads to an increased level of mRNA and proteins and enlargement of cardiomyocytes. **c** Misregulation of CDK9 activity can lead to development of cancers. P-TEFb drives the expression of key pro-survival genes such as *C-MYC* and *MCL-1* (top). Mistargeting of P-TEFb by MYC or MLL-SEC fusion proteins promotes tumour-specific transcriptional amplification (middle). P-TEFb represses tumor-suppressor genes through inhibitory phosphorylation of the BRG1 subunit of the BAF chromatin-remodeling complex

Given the central importance of P‐TEFb in regulating gene expression and maintaining cellular homeostasis, it is perhaps not surprising that CDK9 activity is linked to many pathologic processes. For example, P-TEFb is an essential cellular co-factor required for transcription and replication of the human immunodeficiency virus (HIV), and misregulation of CDK9 activity is associated with cardiac hypertrophy and cancer development [8, 264] (Fig. 6).

### P-TEFb is an essential co-factor of HIV

Viruses often subvert the host cell transcriptional machinery for their own needs. P-TEFb is a primary target of HIV, but is also used by other viruses such as human T‐lymphotropic virus (HTLV‐1), Herpes simplex virus (HSV‐1 and HSV‐2), human cytomegalovirus (CMV), Epstein‐Barr virus (EBV), human adenovirus, Influenza A virus, Dengue virus and Kaposi's sarcoma‐associated virus (KSHV) [264]. Most of the viruses mentioned above hijack P-TEFb via physical interaction with virus-encoded proteins to promote efficient transcription of the viral genome. The discovery that P-TEFb has an essential function in HIV transcription greatly helped to elucidate the molecular mechanisms underlying the replication of the virus. In turn, HIV transcription has become a paradigm for studying P-TEFb function in RNAPII regulation.

The HIV virus integrates into the host genome, and viral gene expression from the Long-Terminal Repeat (LTR) promoter is principally controlled at the early elongation stage by DSIF/NELF-mediated RNAPII pausing [265] (Fig. 6a). Synthesis of full-length HIV transcripts requires expression of a viral transactivator protein, Tat. Activation of elongation primarily relies on Tat-mediated recruitment of P-TEFb to the transactivation response element (TAR) that forms spontaneously at the 5′ end of nascent HIV transcripts [266]. The HIV-encoded Tat protein binds to Cyclin T1 and targets P-TEFb to the TAR RNA structure to stimulate transcription elongation in the same way as it acts on cellular genes, i.e., through phosphorylation of DSIF, NELF and the RNAPII CTD. The crystallographic structure of the P-TEFb/Tat complex indicates that binding of Tat induces a conformational change in CDK9 that may alter its substrate specificity [267]. Of note, only Cyclin T1-containing P-TEFb is targeted by Tat, as the interaction relies on a TRM motif that is not found in cyclin T2. Tat and P-TEFb are recruited to TAR within the SEC to stimulate proviral genome transcription [103, 105] and remain associated with the elongating RNAPII machinery throughout transcription. Thus, the Tat/TAR system illustrates another means of recruiting P-TEFb to the transcription machinery through an RNA tethering system. By recognizing the nascent RNA, P-TEFb is directly placed at the vicinity of the transcription complex, close to its phosphorylation targets, ensuring efficient release of RNAPII pausing.

Interestingly, HIV transcription appears highly sensitive to the level of active CDK9 in the cell [268], making P-TEFb an attractive anti-HIV drug target. Reduced Cyclin T1 levels and hypophosphorylation of CDK9 on Thr186 contribute to HIV latency in primary CD4( +) T cells and these are both substantially up-regulated upon activation [269]. In addition, expression of Tat was shown to significantly increase the cellular pool of available P-TEFb. Tat is capable of disassembling the 7SK RNP by competing with HEXIM1 for binding to both Cyclin T1 and 7SK snRNA [105, 207, 218], and can then capture 7SK-evicted P-TEFb to support efficient viral transactivation (Fig. 6a). In addition, Tat promotes non-degradative ubiquitinylation of HEXIM1 by the UBE2O ubiquitin ligase, which results in cytoplasmic retention of HEXIM1, which may impede reassembly of the 7SK/P-TEFb RNP [225]. Of note, the HTLV‐1 Tax transactivator protein also displaces P-TEFb from the 7SK snRNP, suggesting that 7SK RNP disassembly could be a wide-spread strategy used by viruses to promote viral gene expression [237]. Importantly, Tat and BRD4 form mutually exclusive complexes with P-TEFb as BRD4 interacts with the same region of Cyclin T1 as Tat [240, 250]. Consequently, inhibition of BRD4 favors Tat/P-TEFb interaction, increases HIV transcription and reactivates HIV from latency [270]. Thus, inhibitors of BRD4 such as JQ1 are good candidates to reactivate latent integrated proviruses to facilitate eradication of the viral reservoir. In contrast, CDK9 inhibitors have been shown to be effective in suppressing replication of the virus, but their long-term cytotoxicity remains a serious obstacle to being broadly used in HIV treatment. Targeting the TAR RNA and/or the Tat/P-TEFb interaction surface could be used to inhibit HIV transcription [271]. Additional mechanistic insights into P-TEFb function and regulation may provide the opportunity to develop novel strategies to block RNAPII transcription of the HIV and other viral genomes in the near future.

### P-TEFb and cardiac hypertrophy

Cardiac hypertrophy is an adaptive response of the heart to mechanical overload and pressure, frequently seen in case of hypertension and myocardial infarction. It is characterized by an enlargement of the size of cardiac myocytes as a result of an overall increase in RNA and protein content [272]. P-TEFb appears to play a critical role in this process. Known hypertrophic signals, such as stimulation with endothelin-1 or mechanical stresses, lead to disruption of the 7SK/P-TEFb complex and shift the P-TEFb equilibrium towards the active form [273, 274] (Fig. 6b). The molecular mechanisms that lead to P-TEFb de-repression under hypertrophic conditions remain poorly understood, but likely involve activation of calcium-dependent (calcineurin) and Jak/STAT signaling cascades [273, 274]. Disassembly of the 7SK RNP substantially increases the level of active P-TEFb in cells, where CDK9 activity is normally limiting [275], resulting in enhanced RNAPII transcription, increased mRNA/protein synthesis and cell enlargement. The SEC and BRD4 are implicated in targeting P-TEFb to genes to stimulate RNAPII pause release genome-wide [276, 277] (Fig. 6b). Accordingly, a broad BRD4 redistribution occurs during establishment of the cardiomyocyte hypertrophic gene program, with increased chromatin occupancy at promoters and super-enhancers [277, 278]. Consistent with a stimulation of productive elongation, the level of CTD phosphorylation, and in particular that of Ser2, rises under conditions of hypertrophic stress [274]. Besides globally activating RNAPII elongation, stimulation of CDK9 activity is also accompanied by a specific ‘hypertrophic’ transcriptional reprogramming (Fig. 6b). For instance, CDK9 is specifically targeted to cardiac hypertrophy-responsive promoters through association with important regulators of cardiomyocytes proliferation such as GATA4 and MEF2 [260, 279]. Over-activation of P-TEFb also represses the promoter activity and subsequent expression of PGC-1 (peroxisome proliferator-activated receptor-γ coactivator-1), a master regulator of mitochondrial function and biogenesis [280]. As a consequence, constitutive activation of CDK9 leads to mitochondrial dysfunction, which engenders myocyte apoptosis and predisposes the patient to heart failure. Importantly, recent data indicate that the heart is refractory to Myc-driven transcription due to the low P-TEFb availability in cardiomyocytes [275]. Thus, abnormal P-TEFb activity in heart cells may profoundly reshape RNAPII transcription by allowing abnormal activation by various transcription factors, including Myc.

Control of P-TEFb availability by the 7SK snRNP appears to be an essential mechanism for cardiomyocyte function and development. Accordingly, manifestation of cardiac hypertrophy can be recapitulated by artificial activation of P-TEFb in mice. For instance, the genetic deletion of the HEXIM1 murine homolog, CLP-1 (cardiac lineage protein 1), results in embryonic lethality and mimics the characteristics of cardiac hypertrophy [281]. Similarly, RNAi-mediated inactivation of 7SK RNA in cardiomyocytes induces CDK9 activation and abnormal cell growth, and mice overexpressing Cyclin T1 also exhibit cardiac hypertrophy [274]. Finally, a recent study showed that 7SK destabilization through LARP7 knock-down also increases Ser2P phosphorylation and cardiomyocyte proliferation in zebrafish [282]. Together, these studies demonstrated that CDK9 is a pivotal regulator of the cardiomyocyte transcriptional program, the perturbation of which can lead to cardiomyopathies. Since CDK9 pharmacological inhibitors dampen hypertrophic signals [274], modulation of CDK9 activity may thus be a relevant therapy for some kinds of heart failure [283]. Reducing CDK9 activity to normal baseline levels could theoretically reduce heart growth, reverse the adverse’hypertrophic’ transcriptional program and restore mitochondrial function. From a scientific point of view, cardiac hypertrophy represents an ideal physiopathological model to interrogate the molecular mechanisms that govern sequestration of P-TEFb by 7SK/HEXIM, and may provide insights into the signaling cascades and post-translational modifications that modulate this critical transcriptional regulatory system.

### P-TEFb in cancers

Given that the P-TEFb-dependent transition into productive elongation is an abundantly exploited step in regulating cellular gene expression, it is not surprising that CDK9 contributes to the progression and the maintenance of many cancer types. Accordingly, P-TEFb hyperactivity promotes malignant transformation of fibroblasts in vitro and is associated with hematological, prostate, ovarian, mammary, hepatic, pancreatic and lung cancers [8]. Due to the broad requirement for P-TEFb during embryonic gene expression, genetic alterations affecting the CDK9 or Cyclin T1 loci are relatively rare. Instead, cancers are often associated with increased CDK9 function resulting from modulation of CDK9 association with regulatory proteins and/or increased targeting to genes (Fig. 6c). Cancers are accompanied by a profound transcriptional reprogramming, and a large proportion of cancer cells display widespread effects on mRNA transcription elongation and processing [284].

Importantly, P-TEFb promotes transcriptional elongation of key signal-responsive genes that are themselves critical for proliferation, development and stress responses (Fig. 6c, top panel). Among genes whose expression is highly dependent on CDK9 activity are those encoding MYC, BCL2 (B cell lymphoma 2) and MCL1 (myeloid cell leukemia 1), which have been found to be over-expressed in a wide range of human malignancies and are thought to drive cell survival [8, 285]. Expression of these pro-survival genes is controlled by super-enhancer-mediated transcription, which can be effectively inhibited by CDK9 inhibitors such as Flavopiridol [286]. Super-enhancers activate key oncogenic drivers in many tumor cells [287]. Interestingly, BRD4, the major CDK9 partner, is heavily loaded onto super-enhancers, including that of *C-MYC*, and could help to recruit P-TEFb to super-enhancer-regulated (onco)genes. Accordingly, BRD4 inhibition impairs CDK9 recruitment to *MYC*, reduces *MYC* transcripts levels, down-regulates Myc-dependent target genes, and has anti-proliferative effects in cancer cells [253, 254, 288]. Thus, CDK9 and BRD4 have emerged as druggable targets for the development of cancer therapies, through suppression of constitutive expression of anti-apoptotic proteins.

Of particular significance is the physical interaction of P-TEFb with MYC [58]. c-Myc is a potent oncogene that drives proliferation by globally amplifying gene expression in a P-TEFb-dependent manner [58, 289] (Fig. 6c, middle panel). Mechanistically, Myc-mediated loading of CDK9 to active genes is intensified in cells with high Myc levels, boosting the production of transcripts and resulting in transcriptional amplification. Thus, the interplay between MYC and P-TEFb can contribute to the establishment of tumor-specific gene expression signatures in cancer cells. Incorporation of P-TEFb into the SEC is another source of transcriptional activation in MLL-associated leukemia. As a result of in-frame gene translocations, several components of the SEC, such as AFF1, AFF4, AF9, ELL1 and ENL, form chimeric fusion proteins with the mixed lineage leukemia (MLL) protein [104, 290]. Consequently, SEC can be mis-targeted to MLL-dependent genes, where P-TEFb can release RNAPII into productive elongation in an uncontrolled manner (Fig. 6c).

Malfunction of the 7SK/P-TEFb regulatory machine that abnormally increases P-TEFb activity can also lead to uncontrolled growth. Low expression levels of 7SK RNP components have been associated with various types of human malignancies, including cervical, thyroid, gastric and breast cancer [291–293]. Accordingly, shifting the P-TEFb functional equilibrium towards the active state causes mammary epithelial transformation [202]. Disruption of the P-TEFb functional equilibrium also promotes an epithelial–mesenchymal transition (EMT), invasion and metastasis of breast cancer cells by favoring the P-TEFb-dependent expression of EMT/metastasis-related genes [293]. Finally, frequent frameshift mutations in the *LARP7* gene are found in gastric cancers, pointing to a tumor suppression function for LARP7 [202, 292]. Together, these observations suggest that shifting back the P-TEFb equilibrium towards its inactive form may reduce proliferation and aggressiveness of cancer cells. In line with this, the anti-cancer activities (inhibition of growth, apoptosis) of BRD4 inhibitors have been shown to rely, at least in part, on transcriptional induction of the HEXIM1 gene, which allows recapture of P-TEFb into the 7SK inhibitory complex [294, 295].

Like CDKs in general, CDK9 targeting has been an active area of research in oncology, and a number of CDK9 inhibitors have reached preclinical and clinical development [8]. Interestingly, CDK9 inhibition also reactivates, in multiple cancer cells, tumor-suppressive genes that were epigenetically silenced. Reactivation occurs through BRG1-mediated chromatin remodeling at promoters, and it was proposed that P-TEFb-mediated phosphorylation of BRG1 prevent the BAF complex being recruited to chromatin [164] (Fig. 6c, bottom panel). Thus, the anti-tumoral effects of CDK9 inhibition can be attributed to both suppression of constitutive expression of anti-apoptotic proteins (such as MCL1 and/or MYC) and re-expression of tumor suppressor genes.

## Concluding remarks

Releasing promoter-proximally paused RNAPII into productive elongation by P-TEFb is now recognized as a key regulatory step in gene expression. In the last decade, CDK9 has emerged as a critical coordinator of RNAPII transcription operating at multiple locations throughout the transcription unit. Mechanisms that dictate the duration of the pause and/or regulate loading of P-TEFb onto selected promoters are currently being intensively dissected, since recruitment of active P-TEFb to genes seems to be the rate-limiting step for pause escape. Through coupling pause release with both initiation and termination, CDK9 has a major impact on transcriptional outputs. As such, CDK9 represents an attractive target for cancer therapeutics [8], and there are great hopes that inhibition of CDK9 can be used to effectively and selectively restrict cell growth. The list of CDK9 targets and interactors continues to grow, unveiling new avenues of investigation. It is now critical to fully characterize the transcriptional regulatory networks that are organized around CDK9 to understand how they are regulated during development, stress or signaling-induced activation. The most recent advances have uncovered that phosphatases and termination factors can counteract CDK9 activity to regulate pause duration and finely control gene expression. A particularly important objective is now to fully characterize the complex interplay of P-TEFb and the other transcriptional kinases with phosphatase activities throughout the transcription cycle.

## Data Availability

Not applicable.
